# Complex-Type N-Glycans Influence the Root Hair Landscape of Arabidopsis Seedlings by Altering the Auxin Output

**DOI:** 10.3389/fpls.2021.635714

**Published:** 2021-02-18

**Authors:** Manuel Frank, Heidi Kaulfürst-Soboll, Kerstin Fischer, Antje von Schaewen

**Affiliations:** ^1^Molecular Physiology of Plants, Institute of Plant Biology and Biotechnology, University of Münster (WWU Münster), Münster, Germany; ^2^Department of Molecular Biology and Genetics, Aarhus University, Aarhus C, Denmark

**Keywords:** plant hormones, N-glycosylation, root hairs, auxin, root development

## Abstract

Roots supply plants with nutrients and water, besides anchoring them in the soil. The primary root with its lateral roots constitutes the central skeleton of the root system. In particular, root hairs increase the root surface, which is critical for optimizing uptake efficiency. During root-cell growth and development, many proteins that are components of, e.g., the cell wall and plasma membrane are constitutively transported through the secretory system and become posttranslationally modified. Here, the best-studied posttranslational modification is protein N-glycosylation. While alterations in the attachment/modification of N-glycans within the ER lumen results in severe developmental defects, the impact of Golgi-localized complex N-glycan modification, particularly on root development, has not been studied in detail. We report that impairment of complex-type N-glycosylation results in a differential response to synthetic phytohormones with earlier and increased root-hair elongation. Application of either the cytokinin BAP, the auxin NAA, or the ethylene precursor ACC revealed an interaction of auxin with complex N-glycosylation during root-hair development. Especially in *gntI* mutant seedlings, the early block of complex N-glycan formation resulted in an increased auxin sensitivity. RNA-seq experiments suggest that *gntI* roots have permanently elevated nutrient-, hypoxia-, and defense-stress responses, which might be a consequence of the altered auxin responsiveness.

## Introduction

Roots serve as the central organ to supply a plant with nutrients and water and as anchor in the soil. While the primary root (PR) and lateral roots (LRs) constitute the central skeleton of the root system, root hairs (RHs) in particular increase the root surface and thus are critical for and modulated by nutrient and water supply (Zhang et al., 2018; Vissenberg et al., 2020; Waidmann et al., 2020). During root (and RH) growth and development, many proteins are constitutively transported through the secretory system to reside at the plasma membrane (PM), in the apoplast, or the vacuole (Bassham et al., 2008; Schoenaers et al., 2017). Many of them are targets of posttranslational modifications that take place within the lumen or at the cytosolic face of the organelles that belong to this vesicle-connected endomembrane system. Probably one of the most important of these modifications is protein N-glycosylation, which describes the cotranslational addition of glycans to asparagine residues (Asn, N) in the lumen of the endoplasmic reticulum (ER). This occurs at specific consensus sequences N-X-(S/T) with the X symbolizing any amino acid except proline (Pro, P; Pless and Lennarz, 1977). Many of these sequences can be part of a protein, but not all of them are necessarily used or further modified (micro-heterogeneity), leading to a number of glycoprotein variants that are differently decorated (An et al., 2009; Mariño et al., 2010). As N-glycans are relatively large structures, they play a crucial role for the physicochemical properties (hydration, stability), and thus the shape and function of their glycoproteins (Imperiali and O’Connor, 1999; Wormald and Dwek, 1999; Helenius and Aebi, 2001).

The biosynthesis of N-glycans begins on the cytosolic face of the ER with the addition of N-acetylglucosamine (GlcNAc) to dolichol phosphate (Dol-P), followed by the addition of another GlcNAc and five mannoses (Man) from uridine diphosphate (UDP)-GlcNAc and guanosine diphosphate (GDP)-Man, respectively. The resulting Man5GlcNAc2-PP-Dol switches from the cytosolic to the luminal face of the ER (flip-flop) where the addition of four more Man and three glucose (Glc) residues *via* glycosyltransferases takes place (Abeijon and Hirschberg, 1992; Helenius and Aebi, 2002; Gomord et al., 2010). The oligosaccharide is then transferred from Glc3Man9GlcNAc2-PP-Dol to a nascent protein in the ER lumen by the membrane-bound OLIGOSACCHARYL-TRANSFERASE (OST) complex (Yan and Lennarz, 1999). The central, membrane-integral protein subunit that is part of the active center in the OST complex of eukaryotes is called STAUROSPORIN AND TEMPERATURE SENSITIVE 3 (STT3; Nilsson and Von Heijne, 1993; Yan and Lennarz, 2002). Mammals as well as plants harbor two STT3 isoforms (Gallois et al., 1997; Koiwa et al., 2003; Ruiz-Canada et al., 2009). Besides severe growth defects caused by OST subunit DEFECTIVE GLYCOSYLATION 1 (DGL1; Lerouxel et al., 2005), mutations in Arabidopsis *STT3a* only cause changes in root development and enhanced salt sensitivity (Koiwa et al., 2003). Furthermore, under-glycosylation observed in *stt3a* (but not *stt3b*) mutants led to a malfunction in plant defense by compromising the receptor-like kinase ELONGATION FACTOR RECEPTOR (EFR), but not FLAGELLIN-SENSITIVE 2 (FLS2; Häweker et al., 2010).

Proteins that are modified with high mannose N-glycans undergo quality control in the ER (ERQC). Here, the added N-glycans are highly important, since the three terminal glucosyl residues enable binding to the lectin chaperons calnexin and calreticulin during – sometimes repetitive – folding cycles (Helenius and Aebi, 2004). If a protein is folded correctly, a specific Man residue is clipped by class I ER-MANNOSIDASE (ER-MANI/MNS), which is the last step of N-glycan modification in the ER (Liebminger et al., 2009).

The glycoprotein is then transferred to the *cis*-face of the Golgi apparatus in which its N-glycans are further modified, first by α-MANNOSIDASE I (MANI), resulting in a Man5GlcNAc2 structure (Liebminger et al., 2009; Schoberer and Strasser, 2011). N-ACETYLGLUCOSAMINYLTRANSFERASE I/COMPLEX GLYCAN-LESS 1 (GNTI/CGL1; von Schaewen et al., 1993) then adds a GlcNAc residue to the trimmed 1,3 arm, and Golgi α-MANNOSIDASE II/HYBRID GLYCOSYLATION 1 (MANII/HGL1; Strasser et al., 2006) cleaves two mannosyl residues from the 1,6 arm (Johnson and Chrispeels, 1987; Kaushal et al., 1990; Tezuka et al., 1992). Similar to *stt3a*, *cgl1* (*gntI*), and *hgl1* (*manII*) mutants showed enhanced salt sensitivity, however, due to altered N-glycan maturation (Frank et al., 2008; Kang et al., 2008; Kaulfürst-Soboll et al., 2011). Following GNTI, and prior to the addition of another GlcNAc residue by GNTII to the MANII-trimmed 1,6 arm, the *core* N-glycan structure may be further modified with fucose on the proximal, N-attached GlcNAc by α1,3-FUCOSYLTRANSFERASE (FUCTa/FUT11, FUCTb/FUT12) and xylose on the bisecting Man residue by β1,2-XYLOSYLTRANSFERASE (XYLT; Zeng et al., 1997; Leiter et al., 1999; Strasser et al., 2000; Wilson et al., 2001a; Pagny et al., 2003; Kaulfürst-Soboll et al., 2011), with salt sensitivity also detected for the *fuctab xylt* triple mutant (Kang et al., 2008). Also, the two-terminal GlcNAc residues may be further modified. First with galactose by β1,3-GALACTOSYL-TRANSFERASE (GALT) and then with fucose by α1,4-FUCOSYLTRANSFERASE (FUCTc/FUT13), resulting in the formation of Lewis-a epitopes that were exclusively detected in the *trans*-Golgi network (TGN) and at the PM/apoplast, but not at the tonoplast/vacuoles (Melo et al., 1997; Fitchette et al., 1999; Léonard et al., 2002; Strasser et al., 2007). While a functional relevance has not been reported for Lewis-a epitopes so far (Wilson et al., 2001b; Maeda et al., 2016), a differential subcellular localization of FUCTc proteins from different plant species has recently been demonstrated (Rips et al., 2017).

In numerous developmental processes, including the regulation of PR and LR development, the phytohormones ethylene, cytokinin (CK), and auxin are of central importance (Abeles et al., 1992; El-Showk et al., 2013; Waidmann et al., 2020). Moreover, these hormones have a substantial impact on RH initiation and growth, which is of central importance for the uptake of water and nutrients (Vissenberg et al., 2020). Experiments with increased concentrations of NaCl, KCl, or LiCl revealed an importance of Golgi-based complex N-glycan modification during the salt stress response (Kang et al., 2008). Moreover, an increase in RH length has been described for *gntI/cgl1* seedlings (Frank et al., 2008).

Here we report on the role of complex N-glycan modification for root development/architecture in Arabidopsis. Impairment in the formation of fully matured complex N-glycans resulted in an earlier start of RH elongation and a general increase in RH length. The synthetic phytohormones BAP (CK), NAA (auxin), or ACC (ethylene precursor) resulted in shortening of the PR, changes in LR number, and increased RH length. Intriguingly, loss of Lewis-a formation in *galt* or *fuctc* seedlings also resulted in elongated RH growth. Furthermore, all mutants with altered N-glycan processing in the Golgi apparatus appeared hypersensitive to NAA. RNA-seq data of untreated *gntI* vs. wild-type seedling roots point to an upregulation of nutrient- and hypoxia-related responses (among others) that may be related to an altered hormone homeostasis, especially of auxin and ethylene. Together our findings point to an important role of complex N-glycosylation for proteins involved in root development and/or signaling from post-Golgi compartments, as indicated by the re-programmed nuclear gene expression.

## Materials and Methods

### Plant Material and Growth Conditions

The Columbia-0 (Col-0) ecotype of *Arabidopsis thaliana* was used as the wild type. The following mutant and transgenic Arabidopsis plants were used in this study: *gntI* (*cgl1-2*; Frank et al., 2008), *manII* (*hgl1-1*; Kang et al., 2008; Kaulfürst-Soboll et al., 2011), *galt* (*galt1-1*; Strasser et al., 2007), and *fuctc* (*fuctc-1*; Rips et al., 2017). All genotypes were confirmed by PCR analysis. Back-crossing of *galt1-1* was conducted as described in Supplementary Figure S2. A schematic overview of all alleles used in this study is depicted in Supplementary Figure S7.

Seeds were stratified on petri plates for 2–3 days containing plant growth medium [0.5 MS without vitamins, 0.1% (w/v) sucrose, 0.5 g/L MES, adjusted to pH 5.7 with KOH, 1% agar]. For the mock treatment, diluted EtOH (f.c. 3.4 ‰ v/v) and DMSO (2.8 ‰ v/v) were added after autoclaving. Germination was in vertical position for 3 days in a Percival growth chamber with illumination from the top (Osram LUMILUX cool white L 18 W/840) under LD conditions: 16 h light (150 μmol quanta m^−2^ s^−1^) and 8 h darkness at 23°C. Seedlings of two genotypes plus the wild type were transferred to three unsealed, but tape-fixed vertical plates each (with interchanged positions), containing the same medium plus mixed mock, or hormones as indicated: 15 nM BAP (EtOH), 15 nM NAA (DMSO), or 100 nM ACC (H_2_O) plus the other solvent(s) of the mixed mock. For the NAA series, the DMSO concentration in the mock was adapted to the highest NAA concentration used (120 nM), additionally including the EtOH concentration used before. Plates were cultivated in vertical position for seven more days under LD conditions until PRs, LRs, and RHs were measured and counted.

### Plant Transformation and Confocal Laser Scanning (CLSM) Microscopy

The auxin signaling reporter *35S::DII-VENUS* (Brunoud et al., 2012) and the CK signaling reporter *TCSn::GFP* (Zürcher et al., 2013) were introduced into Col-0 wild-type and *gntI* plants *via Agrobacterium tumefaciens*-mediated T-DNA insertion (Logemann et al., 2006). Several homozygous T3 lines were cultivated as stated before. Roots of 3-day-old or 10-day-old seedlings were stained for 5 min at room temperature in a 10 μg ml^−1^ propidium iodide solution (stock: 10 mg ml^−1^ in distilled water), briefly rinsed in distilled water, and analyzed with a Leica TCS SP5 microscope with inverse optics (at 488 nm excitation). Processing of the digital images was accomplished with the program Leica LAS AF.

### Immunoblot Analysis

Immunoblot analyses were conducted essentially as described in Rips et al. (2017). Frozen samples (20 seedlings per genotype, stored at −20°C) were extracted in 120 μl of protein extraction buffer [50 mM HEPES pH 7, 2 mM Na_2_S_2_O_5_, 1:100 (v/v) Proteinase-Inhibitor-Cocktail for use with plant extracts (Sigma), 1 mM Pefabloc SC, 1:100 (v/v) Mercaptoethanol] using a potter and centrifuged at 13,500 rpm (4°C) for 10 min in a table top microfuge. The supernatants were transferred to new tubes and volume equivalents of 25 μg protein each (Bradford assay) were separated by SDS-PAGE on 10% gels and blotted to nitrocellulose membranes (PROTRAN 0.45 μm, GE Healthcare). The pellets were resuspended in 40 μl of protein extraction buffer additionally containing 250 mM NaCl and 0.1% SDS. After incubation for 30 min at room temperature and centrifugation at 13,500 rpm (20°C) for 10 min in a table top microfuge, the same volume as used for the supernatant fractions was separated by SDS-PAGE and blotted. Blots were stained with Ponceau-S solution (Sigma), scanned, de-stained with TBST, and blocked with 5% (w/v) milk powder in TBST overnight at room temperature. Chemiluminescent development with complex glycan antisera was conducted as follows: α-PHA-L (Kaulfürst-Soboll et al., 2011) diluted 1:15,000 or α-HRP (Sigma) diluted 1:50,000 in TBST with milk powder, followed by Goat-anti Rabbit-HRP-conjugate diluted 1:15,000 (Bio-Rad, Munich). JIM84 (rat monoclonal α-Lewis a antibodies; Rips et al., 2017) diluted 1:40 in TBST without milk powder, followed by Goat-anti Rat-HRP-conjugate (Bio-Rad, Munich), diluted 1:15,000 in TBST without milk powder, prior to soaking membranes in ECL Select™ western blotting detection reagent (Amersham/GE Healthcare) and signal recording in a sensitive bioimager (MicroChemi, DNR).

### Primary Root, Lateral Root, and Root Hair Measurement/Counting

After cultivation, vertical plates were scanned, and LRs were counted manually. Root tips of PRs were photographed with a binocular (Model LEICA MZ 16 F with inverse illumination and camera LEICA DFC420 C), as well as RHs (within 6.7 mm, starting from the first visible RH bulb) were measured and counted using ImageJ®. A partially elongated RH was defined as being visibly longer than broad. LR and RH density was calculated using Microsoft Excel®.

### Statistical Analysis

R Studio® was used for statistical analysis of all PR-, LR-, and RH-related data.

Homogeneity and homoscedasticity were tested by Shapiro-Wilk and Levene tests (*p* ≥ 0.05) before ANOVA was performed, followed by a Tukey *post hoc* test. If assumptions were not met, transformations (log, sqrt) were conducted. A Kruskal-Wallis test was performed and corrected for multiple comparisons by running the Benjamini-Hochberg (BH) procedure, if assumptions were still not met after transformation.

### Sampling and RNA Isolation for Transcriptome Analysis by RNA-Seq

For RNA-seq experiments, three *gntI* and wild-type pools, consisting of 25 roots each, were harvested from the vertical plates and total RNA was isolated as described in Frank et al. (2020). After library construction, RNA-seq data were generated as described in Mittal et al. (2017).

### Acquisition and Analysis of RNA-Seq Data

RNA-seq data analysis was performed using the Galaxy platform.^1^ Reads were trimmed using Trimmomatic (Bolger et al., 2014) and quality control performed with FastQC.^2^ Reads were aligned to the TAIR10 reference genome using HISAT2 (Kim et al., 2015) and aligned reads were counted with HTSeq (Anders et al., 2015). Differentially expressed genes (DEG) were extracted using DESeq2 (with BH corrected *p* ≤ 0.05; Love et al., 2014). GO enrichment of DEG was performed using PANTHER^3^ with the binomial test type and Bonferroni correction for multiple testing. Raw data are deposited at the Sequence Read Archive (SRA^4^) with the BioProject ID PRJNA682418.

## Results

### Plants Impaired in Complex-Type N-Glycan Maturation Display Alterations in the Root Hair Landscape

**Figure 1:**
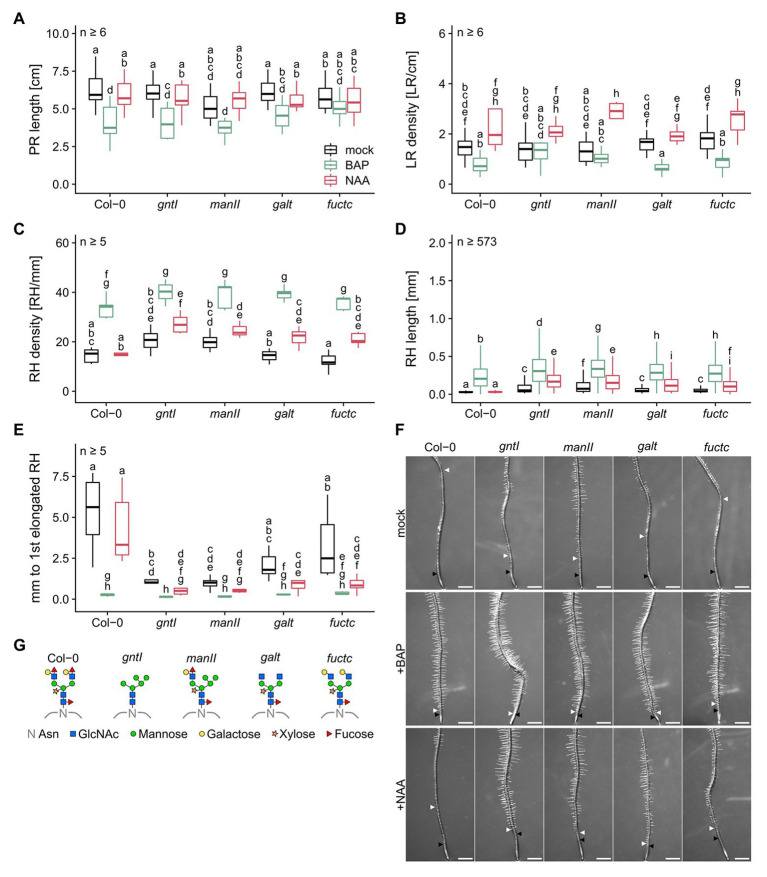
Plants lacking fully matured complex N-glycans differ in the root hair landscape and are auxin hypersensitive. **(A)** Primary root length, **(B)** lateral root density, **(C)** root hair density, **(D)** root hair length, and **(E)** distance between first general and first partially elongated root hair of N-glycosylation mutants and wild-type plants cultivated for 8 days on mock (black boxes), 15 nM BAP (green boxes), or 15 nM NAA (red boxes). Letters indicate significant differences between groups [*p* ≤ 0.05; **(A–C,E)** two-way ANOVA with Tukey *post hoc* test; **(D)** Kruskal-Wallis test, FDR corrected *via* Benjamini-Hochberg]. **(F)** Pictures of wild type (Col-0) and N-glycosylation mutant root tips. Black arrows indicate the first general root hair while white arrows point to the first partially elongated root hair. Scale bars: 500 μm. **(G)** N-glycan structures of the mutants tested. PR, primary root; LR, lateral root; RH, root hair; Asn, Asparagine; GlcNAc, N-acetylglucosamine.

Previous studies by Kang et al. (2008) and Frank et al. (2008) reported that especially under salt stress, but also under normal conditions, absence of complex-type N-glycosylation had an impact on root development. We, therefore, cultivated Col-0 wild type in parallel with *gntI*, *manII*, *galt*, and *fuctc* seedlings to specify the role of N-glycan modification in the Golgi in this context. Immunoblot analysis indicated that only Col-0 and *manII* display binding to Le^a^ epitopes. As published before, *core* fucose- or xylose-specific signals were absent from *gntI*. In *manII*, only little was detected with α-PHA-L vs. α-HRP (Supplementary Figure S1, due to altered binding specificities; Kaulfürst-Soboll et al., 2011), whereas *galt* and *fuctc* behaved similar to wild type. No significant differences in PR length, LR and RH density were observed between the genotypes (Figures 1A–C), but alterations in complex N-glycan modification resulted in an increase of RH length and in a reduced distance between the first general and the first elongated RH (Figures 1D,E). We stained RHs of the wild type and the *gntI* mutant and found that the first RH is initiated at about the same position (ca. 1 mm from the tip), notably with no obvious differences in the meristematic zone (Supplementary Figure S2). Especially *gntI* and *manII* were strongly affected, but also loss of Lewis-a formation in *galt* and *fuctc* resulted in an about two-fold increase of RH length (Col-0: 0.034 vs. *galt* or *fuctc*: 0.066 mm) and an over 40% decrease in the distance between the first general and the first elongated RH compared to wild type. We initially noticed that the roots of some *galt* plants formed very short RHs, which was likely caused by a secondary mutation, since it could be successfully crossed out (Supplementary Figure S3).

Taken together, an impairment in complex-type N-glycan maturation in the Golgi apparatus led to the formation of longer RHs and an earlier start of RH elongation under control conditions.

### Complex-Type N-Glycosylation Dependent Changes in Root Hair Development May Be the Result of an Auxin Imbalance

Cytokinin, auxin, and ethylene are well-studied main regulators of root architecture, and positively influence RH development and elongation (Vissenberg et al., 2020). To examine a possible impact of these hormones on the observed phenotypic alterations in mutants affected in complex N-glycan modification (cgly mutants), we applied either the CK derivative BAP (15 nM), the auxin derivative NAA (15 nM), or the ethylene precursor ACC (100 nM) to 3-day-old seedlings and evaluated the root responses of the mutant genotypes to that of the wild type.

BAP treatment led to a general decrease in PR length, resulted in a mild but mostly not significant reduction in LR density, and was paralleled by an increased RH density with no significant differences among the genotypes (Figures 1A–C). RHs were between three- and seven-times longer compared to control conditions, with the same tendency of longer RHs in the cgly mutants compared to the wild type (e.g., Col-0: 0.034 vs. 0.229 mm or *manII*: 0.118 vs. 0.343 mm; Figure 1D). Strikingly, the differences observed regarding the distance from the first overall to the first elongated RH among the mutant genotypes were completely abolished by the BAP treatment (Figure 1E).

NAA treatment resulted in minor changes of PR length and led to an increased LR density without any significant differences between the genotypes at 15 nM (Figures 1A,B). However, all cgly mutants displayed a hypersensitivity to NAA in terms of RH density, RH length, and distance between the first overall and the first elongated RH (Figures 1C–E). While the wild type did not respond to the NAA treatment, the cgly mutants displayed an up to twice as dense RH system, in which RHs were three to six times longer (e.g., *gntI*: 0.086 vs. 0.192 mm or *fuctc*: 0.066 vs. 0.122 mm; Figure 1D). Moreover, the distance between the first overall and the first elongated RH was reduced up to 7.5 times.

**Figure 2:**
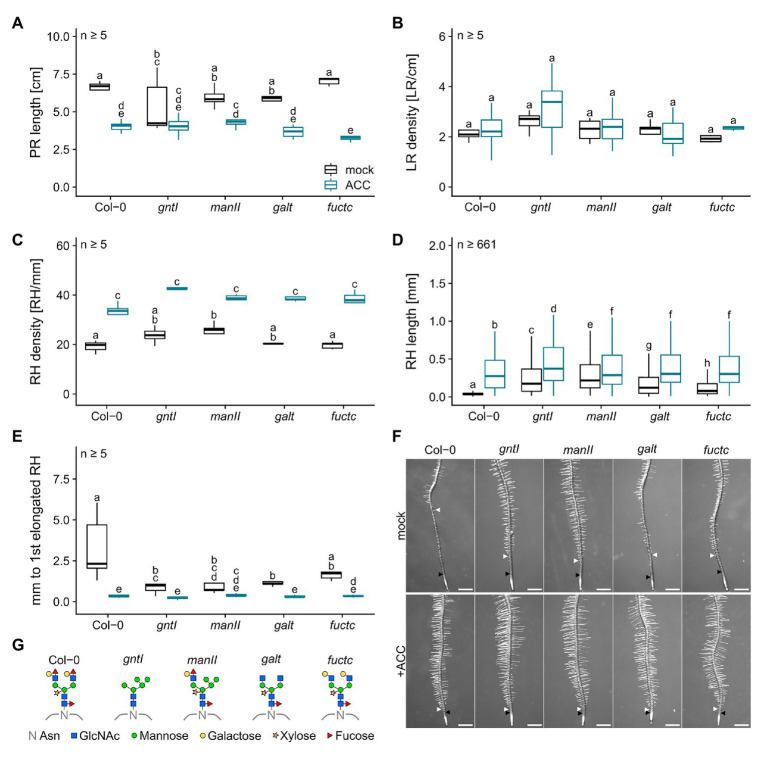
Roots compromised in complex N-glycan modification respond wild type-like to ACC. **(A)** Primary root length, **(B)** lateral root density, **(C)** root hair density, **(D)** root hair length, and **(E)** distance between first general and first partially elongated root hair of N-glycosylation mutants and wild-type plants cultivated for 8 days on mock (black boxes) or 100 nM ACC (blue boxes). Letters indicate significant differences between groups [*p* ≤ 0.05; **(A–C,E)** two-way ANOVA with Tukey *post hoc* test; **(D)** Kruskal-Wallis test, FDR corrected *via* Benjamini-Hochberg]. **(F)** Pictures of wild type (Col-0) and N-glycosylation mutant root tips. Black arrows indicate the first general root hair while white arrows point to the first partially elongated root hair. Scale bars: 500 μm. **(G)** N-glycan structures of the mutants tested. PR, primary root; LR, lateral root; RH, root hair; Asn, Asparagine; GlcNAc, N-acetylglucosamine.

ACC treatment led to a general decrease in PR length, did not impact LR density, and resulted in an increased RH density (Figures 2A–C). RHs were up to three times longer compared to control conditions with the same tendency of longer RHs in the cgly mutants compared to the wild type (e.g., Col-0: 0.064 vs. 0.305 mm or *manII*: 0.284 vs. 0.364 mm; Figure 2D). Notably, differences in the distance from the first overall to the first elongated RH were completely abolished by the ACC treatment (Figure 2E).

**Figure 3:**
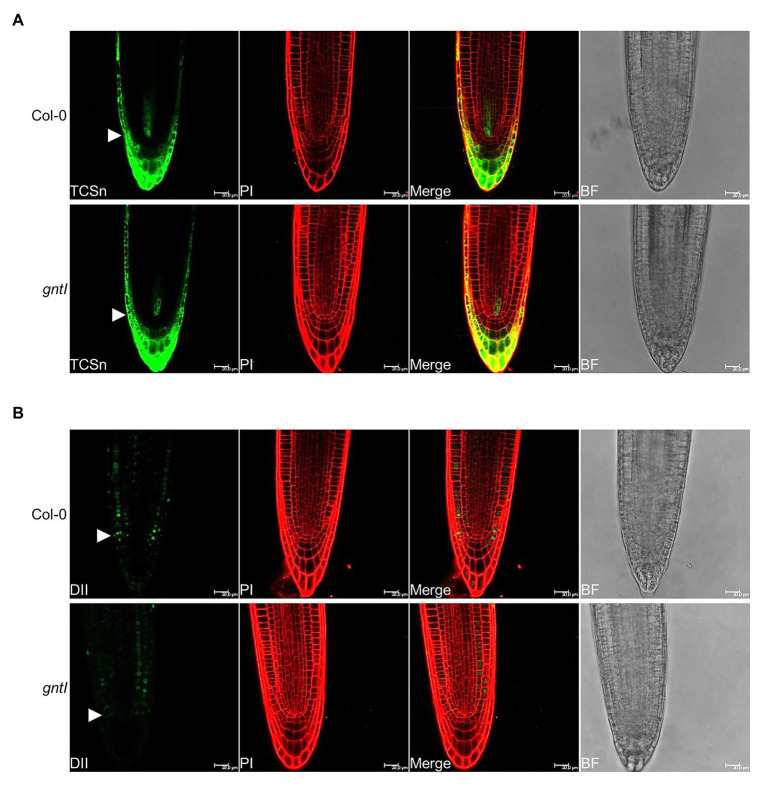
Roots compromised in complex N-glycan modification display a wild type-like CK and an increased auxin signaling output. Pictures of the representative **(A)** TCSn::GFP (TCSn) and **(B)** 35S::DII-VENUS (DII) output in Col-0 and gntI root tips are depicted. White arrows point to the level of the quiescence center. Scale bar: 20 μm. PI, propidium iodide; BF, bright field. More pictures of multiple independent lines can be found in Supplementary Figures S4, S5.

To further support the obtained effects with CK and NAA, we studied the signaling output of these hormones by using reporter constructs. The CK signaling reporter *TCSn::GFP* (Zürcher et al., 2013) did not indicate any differences in GFP signal intensity between Col-0 and *gntI* in various independent transgenic lines (Figure 3A; Supplementary Figure S4), indicating that perhaps minor but no significant differences in CK signaling occur in the cgly mutants. An auxin hypersensitivity of *gntI* root tips was indicated by the reduced fluorescent signals of the *35S::DII-VENUS* reporter (Brunoud et al., 2012) compared to the wild type in various independent lines (Figure 3B; Supplementary Figure S5).

**Figure 4:**
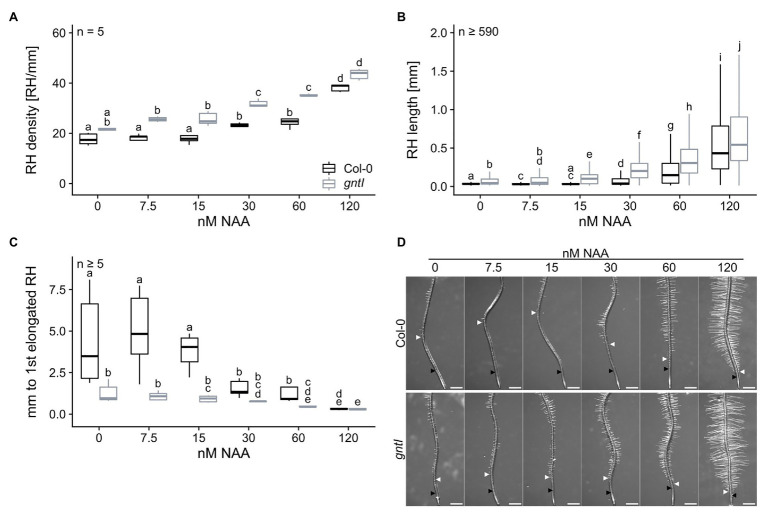
Plants lacking complex N-glycans display an altered auxin response. **(A)** root hair density, **(B)** root hair length, and **(C)** distance between first general and first partially elongated root hair of wild-type (Col-0, black boxes) and gntI plants (gray boxes) cultivated for 8 days on plates supplemented with the indicated NAA concentrations. Letters indicate significant differences between groups [*p* ≤ 0.05; **(A,C)** two-way ANOVA with Tukey *post hoc* test; **(B)** Kruskal-Wallis test, FDR corrected *via* Benjamini-Hochberg]. **(D)** Pictures of representative root tips. Black arrows indicate the first general root hair while white arrows point to the first partially elongated root hair. Scale bars: 500 μm.

Since mostly auxin signaling and responsiveness seemed to be affected in the cgly mutants, we applied NAA in concentrations between 0 and 120 nM to the growth medium to examine the phenotypical response of *gntI* and wild type. NAA application resulted in a concentration-dependent increase of RH density in roots of both genotypes, especially on NAA concentrations ≥ 30 nM (Figure 4A). While *gntI* roots displayed an increased RH density throughout most concentrations compared to wild type, no significant differences in RH density were recorded at 0 and 120 nM NAA. Similarly, RH length increased most dramatically by the application of more than 30 nM NAA (Figure 4B). However, *gntI* roots also displayed significantly longer RHs growing on 15 nM NAA, while wild-type roots were not responsive to this NAA concentration (see also Figure 1D). Lastly, the distance between the first RH bulb and the first partially elongated RH decreased with increasing NAA concentration in both genotypes (Figure 4C; Supplementary Figure S6). While the distance between these RH areas was shorter in *gntI* compared to wild-type roots at most NAA concentrations, it was similar at 120 nM.

Taken together, PR length, LR density, and RH length were affected similarly in the cgly mutants, while differences in the start of RH elongation disappeared by application of the hormones. Interestingly, all mutant genotypes displayed a hypersensitive response to the synthetic auxin NAA in terms of RH length. Of note, abrogation of complex N-glycan modification in *gntI* appeared to increase the auxin-signaling output in root tips, leading to a shift in auxin sensitivity on RH elongation starting around 15 nM NAA.

### Roots Lacking Complex-Type N-Glycosylation Display Mostly Nutrient- and Hypoxia-Related Transcriptional Changes

Apart from studying the response of cgly mutant roots to several phytohormones, we compared the transcriptome of *gntI* and wild-type roots under control conditions. A total of 290 genes appeared to be differentially expressed. Of these, 186 genes were more abundant in *gntI* while 104 were less abundant. Among these genes, At4g33880, coding for the bHLH-VIIIc-/RSL-type transcription factor *ROOT HAIR-DEFECTIVE 6-LIKE 2* (*RSL2*), which is involved in auxin-dependent RH elongation (Yi et al., 2010; Bhosale et al., 2018), was about two-fold more abundant in *gntI* (Supplementary Table S1). GO-term enrichment for biological processes revealed that those linked to nitrate metabolism and hypoxia/oxygen deprivation are significantly over-represented in *gntI* (Table 1). Moreover, enrichment for the GO term “molecular function” showed that those related to oxygen were over-represented as well (Table 2). Aside defense-related genes, GO terms directly connected to hormone homeostasis or signaling were not significantly over-/under-represented. But genes that are part of the GO term “cellular response to hypoxia” were found among the strongest DEG, notably in the ethylene-dependent hypoxia response (Supplementary Table S1; Yang et al., 2011).

**Table 1:** Enrichment of GO terms related to biological processes of differentially expressed genes between *gntI* and wild-type seedling roots.

GO term	GO ID	Over(+)/under(−) represented	Fold enrichment	*p*-value
Nitrate assimilation	0042128	+	36.52	1.54E-02
Nitrate metabolic process	0042126	+	36.52	1.54E-02
Reactive nitrogen species metabolic process	2001057	+	28.69	3.94E-02
Cellular response to hypoxia	0071456	+	5.56	2.74E-03
Defense response, incompatible interaction	0009814	+	5.55	5.00E-02
Cellular response to decreased oxygen levels	0036294	+	5.51	3.01E-03
Cellular response to oxygen levels	0071453	+	5.49	3.15E-03
Response to hypoxia	0001666	+	5	8.54E-03
Response to decreased oxygen levels	0036293	+	4.93	1.00E-02
Response to oxygen levels	0070482	+	4.89	1.09E-02
Innate immune response	0045087	+	4.59	9.38E-03
Immune response	0006955	+	4.49	1.21E-02
Defense response to bacterium	0042742	+	4.37	7.70E-04
Immune system process	0002376	+	3.99	4.45E-02
Response to bacterium	0009617	+	3.95	7.86E-04
Defense response to other organism	0098542	+	3.48	4.34E-05
Defense response	0006952	+	3.48	2.13E-06
Response to external biotic stimulus	0043207	+	3.02	6.31E-05
Response to other organism	0051707	+	3.02	6.31E-05
Response to biotic stimulus	0009607	+	3.02	6.45E-05
Interspecies interaction between organisms	0044419	+	2.95	1.14E-04
Response to external stimulus	0009605	+	2.46	1.47E-03
Response to stress	0006950	+	2.3	4.34E-08
Response to stimulus	0050896	+	1.75	1.26E-05

**Table 2:** Enrichment of GO terms related to molecular functions of differentially expressed genes between *gntI* and wild-type seedling roots.

GO term	GO ID	Over(+)/under(−) represented	Fold enrichment	*p*-value
Manganese ion binding	0030145	+	11.37	3.24E-02
FAD binding	0071949	+	9.25	2.43E-02
Flavin adenine dinucleotide binding	0050660	+	6.4	9.21E-03
Heme binding	0020037	+	4.83	1.09E-04
Iron ion binding	0005506	+	4.63	2.33E-03
Tetrapyrrole binding	0046906	+	4.47	3.36E-04
Oxidoreductase activity, acting on paired donors, with incorporation or reduction of molecular oxygen	0016705	+	4.36	1.07E-03
Transition metal ion binding	0046914	+	2.98	1.91E-05
Oxidoreductase activity	0016491	+	2.89	1.34E-06
Metal ion binding	0046872	+	2.5	1.96E-04
Cation binding	0043169	+	2.45	3.02E-04
Ion binding	0043167	+	2.29	2.71E-07
Catalytic activity	0003824	+	1.53	2.06E-05

All in all, the genome-wide transcriptome analysis revealed that a lack of complex-type N-glycans might induce RH elongation by increasing the abundance of genes known to be induced by auxin and/or ethylene.

## Discussion

### Proteins Bearing Complex-Type N-Glycans May Be Involved in Root Hair Elongation

A fully developed root system is crucial to anchor a plant in the soil and to enable sufficient nutrient uptake. Our study demonstrated that any impairment in the structure of complex N-glycans – including the loss of Lewis^a^ epitopes – results in alterations of the RH system, characterized by an earlier and more pronounced RH elongation (Figure 1). So far, an increased RH system has been described for the complete loss of complex-type N-glycosylation, e.g., in *gntI* seedlings (Frank et al., 2008; Liebminger et al., 2009). The apparent importance of Lewis^a^ epitopes for RH elongation displays a novelty, since no other developmental process in which Lewis^a^ epitopes are of particular relevance has been identified so far. During RH elongation, the cell wall at the RH tip is periodically acidified and loosened, and new cell-wall components for the PM and apoplast are constantly synthesized in the ER, modified in the Golgi apparatus, and secreted (Balcerowicz et al., 2015; Schoenaers et al., 2017). Especially glycoproteins that depend on complex-type N-glycan modification seem to be compromised in Arabidopsis and rice *gntI* mutants, leading to a reduced cellulose content (Kang et al., 2008; Fanata et al., 2013). Moreover, impairments in complex N-glycosylation affect the function and stability of KORRIGAN1, a glycoprotein with eight mostly complex N-glycans (Kang et al., 2008; Liebminger et al., 2013; Rips et al., 2014; Nagashima et al., 2020; Kaulfürst-Soboll et al., 2021), which plays an important role for cellulose synthesis at the PM (Nicol et al., 1998; Sato et al., 2001).

Of course, in the cgly mutants, several glycoproteins that are transported to the PM can be affected and thus (indirectly) affect RH elongation. The RNA-seq results described in this study hint at only mild changes of the transcriptome caused by missing complex N-glycan formation in *gntI* and were mostly related to hypoxia and nitrate responses (Tables 1, 2). They include increased abundance of *RSL2* in *gntI*, which might contribute to its longer RHs, even though overexpression of *RSL2* did not result in an exaggerated RH elongation (Yi et al., 2010).

All in all, the RNA-seq results suggest a stronger impact of missing complex N-glycan modification on the protein level, regarding characteristics like glycoprotein stability, protein interaction, substrate binding, and/or enzyme kinetics.

### Complex N-Glycosylation Dependent Changes in the Root Hair Landscape Might Be the Result of Altered Hormone Signaling and/or Homeostasis

Phytohormones have a substantial impact on PR and LR development, as well as RH initiation and growth, which are all essential for water and nutrient uptake (Vissenberg et al., 2020; Waidmann et al., 2020). BAP (synthetic CK) and ACC (ethylene precursor) application experiments performed in this study revealed that PR length, LR density, and RH length are affected similarly in the cgly mutants vs. wild type, while differences in the start of RH elongation disappeared by application of these hormones (Figures 1, 2). Also, the *TCSn::GFP* reporter did not indicate major differences in CK signaling between Col-0 and *gntI* (Figure 3A; Supplementary Figure S4). These results contrast studies reporting a CK hyposensitivity of the rice *gntI* mutant (Fanata et al., 2013). In principle, CK output may be influenced by complex N-glycosylation at various levels. For example, CK perception by ARABIDOPSIS HISTIDINE KINASEs (AHKs) takes place in part at the PM (Antoniadi et al., 2020; Kubiasová et al., 2020), and these receptors harbor potential N-glycosylation sites in their ectodomains (e.g., AHK4/CRE1/WOL1). At least the localization and thus function of CYTOKININ INDEPENDENT1 (CKI1), another histidine kinase involved in CK perception (Kakimoto, 1996; Deng et al., 2010), was sensitive to the complete absence of N-glycans as shown by tunicamycin treatment (Hwang and Sheen, 2001). Also, CK transport *via* AZA-GUANINE RESISTANT1 (AZG1) and AZG2, which were localized at the ER and the PM (Tessi et al., 2020), could be affected, since these transport proteins may harbor N-glycans as well. In contrast, ethylene signaling components are confined to the ER and therefore unlikely to be direct targets of complex N-glycan modification. Nevertheless, the transcriptome data for *gntI* seedling roots indicate that ethylene-dependent hypoxia responses are impacted (Table 1; Supplementary Table S1; Yang et al., 2011).

Apart from CK and ethylene, all cgly mutants were revealed to be auxin-sensitive, particularly in terms of RH length. Application of increasing NAA concentrations to wild-type and *gntI* roots demonstrated that auxin sensitivity in *gntI* is altered as much as it is mimicked by the perception of 15 nM NAA in wild type (Figures 1, 4), potentially impacting crosstalk between auxin and ethylene in roots (Swarup et al., 2007). Supportive for an altered auxin status was the increased auxin signaling output in *gntI* root tips compared to wild type using the DII-VENUS reporter (Figure 3B; Supplementary Figure S5). The latter would explain the transcriptome changes in *gntI*, since auxin signaling is increased in root tips under hypoxia (Eysholdt-Derzsó and Sauter, 2017), and ethylene-responsive genes were enriched among the GO term “cellular response to hypoxia” (Supplementary Table S1). Moreover, *RSL2* expression is controlled by auxin signaling (Bhosale et al., 2018).

Deficiency of complex N-glycan modification could affect the auxin output at different levels. Potential targets within the auxin-signaling cascade may involve the family of TRANSMEMBRANE KINASE (TMK) receptor-like kinases (Xu et al., 2014). For example, TMK1 harbors six potential N-glycosylation sites (Chang et al., 1992). A parallel study reports on altered auxin signaling for *GNTI*-RNAi and *MANII*-RNAi tomato lines during fruit ripening (Kaulfürst-Soboll et al., 2021), and especially free mannose-terminated N-glycans of Man_5_GlcNAc structure were found to antagonize auxin-induced fruit ripening in tomato (Yunovitz and Gross, 1994). One could therefore speculate that the occurrence, lifetime, or activity of free N-glycans differs in wild type compared to roots of the cgly mutants, and thus results in altered auxin responsiveness. Another potential target could be auxin transport by PINOID (PIN) or ATP-BINDING CASSETTE B (ABCB) proteins, which harbor N-glycosylation sites as well, e.g., the *abcb4* mutant displays shorter RHs than Col-0 wild type (Kubeš et al., 2011). Supporting this, previous studies suggest that auxin import *via* ABCBs requires complex-type N-glycans to positively regulate RH growth in rice (Wang et al., 2014) and that auxin transport is impaired in rice shoots lacking *core* fucosylation (Harmoko et al., 2016).

**Figure 5:**
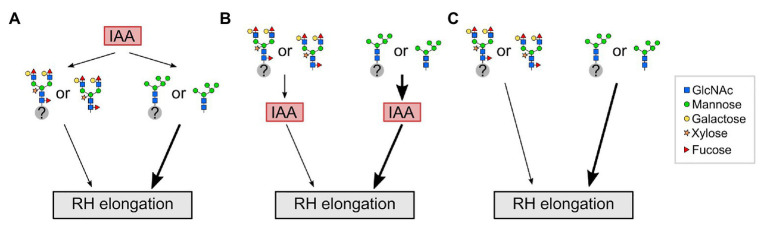
Potential interplay between auxin (IAA) and complex N-glycosylation during RH elongation. **(A)** In wild-type roots with fully matured complex N-glycans, a (group of) yet unknown N-glycosylated factor(s) (gray circle with question mark) or free N-glycans act downstream of auxin (indole acetic acid, IAA) to regulate RH elongation. An impairment of fully matured N-glycans in complex glycosylation mutants (depicted by N-glycans as they appear in *gntI*) results in exaggerated elongation of RHs (thick black arrow). **(B)** Complex N-glycosylation acts upstream of IAA to regulate RH elongation. An impairment of fully matured N-glycans in complex glycosylation mutants results in exaggerated RH elongation by enforcing the auxin output. **(C)** Complex N-glycosylation acts independent of IAA on RH elongation. RH, root hair; GlcNAc, N-acetylglucosamine.

Based on the results of this study, we propose a model in which the influence of complex-type N-glycans on RH elongation may lie either upstream or downstream of the auxin output (Figure 5). For example, auxin transporters may be affected differently by immature vs. fully matured N-glycans, which could be downstream of indole acetic acid (IAA; panel A), while the scenario of a receptor with altered signaling output is depicted in panel B, and alterations operating in parallel to hormone signaling in panel C. We speculate that in wild-type roots with fully matured complex N-glycans, a (group of) yet unknown N-glycosylated factor(s) or free N-glycans regulate(s) RH elongation. An impairment of fully matured complex N-glycans in the cgly mutants enforces the auxin output through these factors, resulting in exaggerated RH elongation. To identify one of the key players will be a challenge for future experiments.

## Data Availability Statement

The original contributions presented in the study are publicly available. This data can be found here: https://www.ncbi.nlm.nih.gov/bioproject/PRJNA682418.

## Author Contributions

MF and AvS developed the project. MF, AvS, HK-S, and KF designed and performed experiments. MF, HK-S, and AvS analyzed data. MF and AvS wrote the article with final contributions of HK-S. All authors contributed to the article and approved the submitted version.

### Conflict of Interest

The authors declare that the research was conducted in the absence of any commercial or financial relationships that could be construed as a potential conflict of interest.

## Footnotes

^1^ https://usegalaxy.eu/

^2^ http://www.bioinformatics.babraham.ac.uk/projects/fastqc/

^3^ http://pantherdb.org/

^4^ http://www.ncbi.nlm.nih.gov/bioproject/682418
