# Erratum: Complex-Type N-Glycans Influence the Root Hair Landscape of Arabidopsis Seedlings by Altering the Auxin Output

**DOI:** 10.3389/fpls.2021.811353

**Published:** 2021-11-30

**Authors:** Frontiers Production Office

**Affiliations:** Frontiers Media SA, Lausanne, Switzerland

**Keywords:** plant hormones, N-glycosylation, root hairs, auxin, root development

Owing to a production error, in the *Plant Material and Growth Conditions* subsection of the *Materials and Methods*, the value for BAP concentration was presented incorrectly as 0.15 nM in the following sentence:

“Seedlings of two genotypes plus the wild type were transferred to three unsealed, but tape-fixed vertical plates each (with interchanged positions), containing the same medium plus mixed mock, or hormones as indicated: 0.15 nM BAP (EtOH), 15 nM NAA (DMSO), or 100 nM ACC (H_2_O) plus the other solvent(s) of the mixed mock.”

The corrected sentence with the correct BAP concentration (15 nM) is as follows:

“Seedlings of two genotypes plus the wild type were transferred to three unsealed, but tape-fixed vertical plates each (with interchanged positions), containing the same medium plus mixed mock, or hormones as indicated: 15 nM BAP (EtOH), 15 nM NAA (DMSO), or 100 nM ACC (H_2_O) plus the other solvent(s) of the mixed mock.”

The publisher apologizes for this mistake. The original article has been updated.

