# Supplementary material for: Complex-Type N-Glycans Influence the Root Hair Landscape of Arabidopsis Seedlings by Altering the Auxin Output
Source: Front Plant Sci. 2021 Feb 18;12:635714. doi: 10.3389/fpls.2021.635714 (PMC7930818; doi:10.3389/fpls.2021.635714)
Supplement: Supplementary file 1 [file Data_Sheet_1.zip › Data_Sheet_1.pdf]

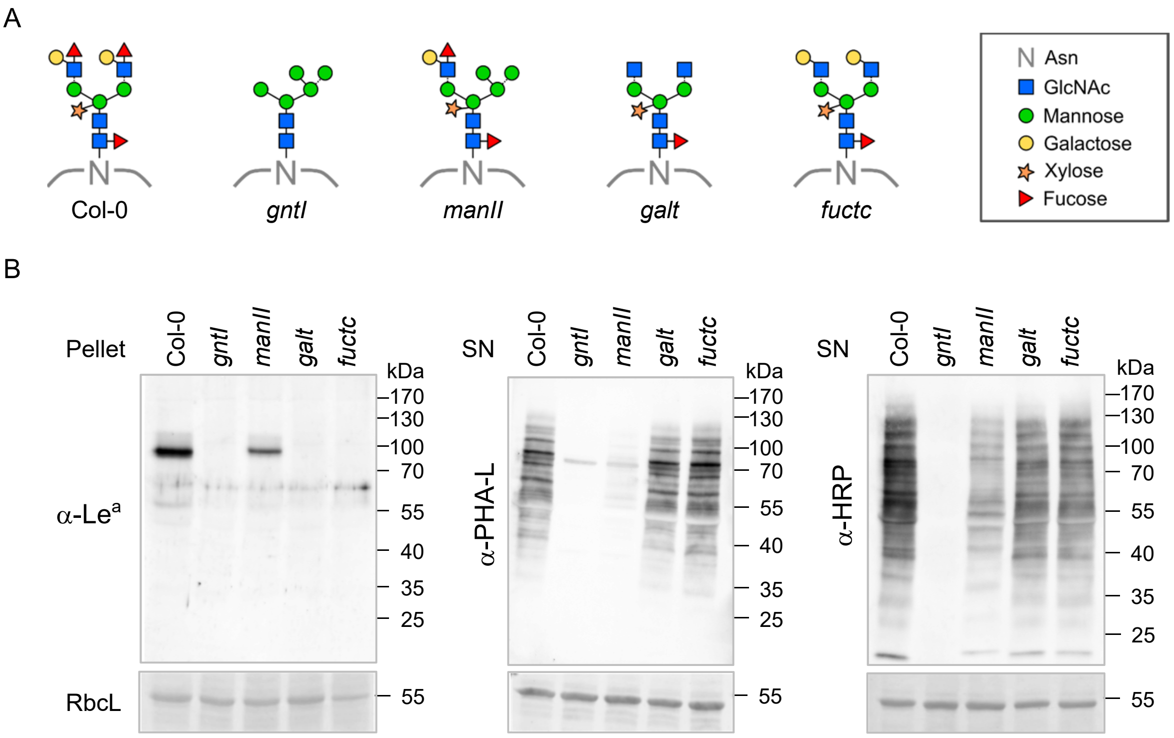

Figure S1. Immunoblot analyses of Arabidopsis seedlings (prior to transfer). A) Major N-glycans produced in the indicated genotypes. B) Arabidopsis seedlings (germinated for 3 d) were harvested (20 each) and frozen. Total soluble proteins were extracted without SDS (supernatant fractions, SN). Pellets (containing insoluble proteins) were then extracted in 3x lower buffer volume with 0.5% SDS. Lewis-a signals (α-Le^a, JIM84) were detected mainly in the pellet fractions, complex N-glycan signals were similarly abundant in both fractions (here, SN fractions). Note that α-HRP recognizes both xylose and core fucose, whereas α-PHA-L is largely xylose-specific. The prominent band of RubisCO large subunit (RbcL) of the Ponceau S-stained blots is shown as loading reference. Apparent molecular masses are indicated in kDa (PageRuler Prestained Protein Ladder, Fermentas). Asn, Asparagine; GlcNAc, N-acetylglucosamine.

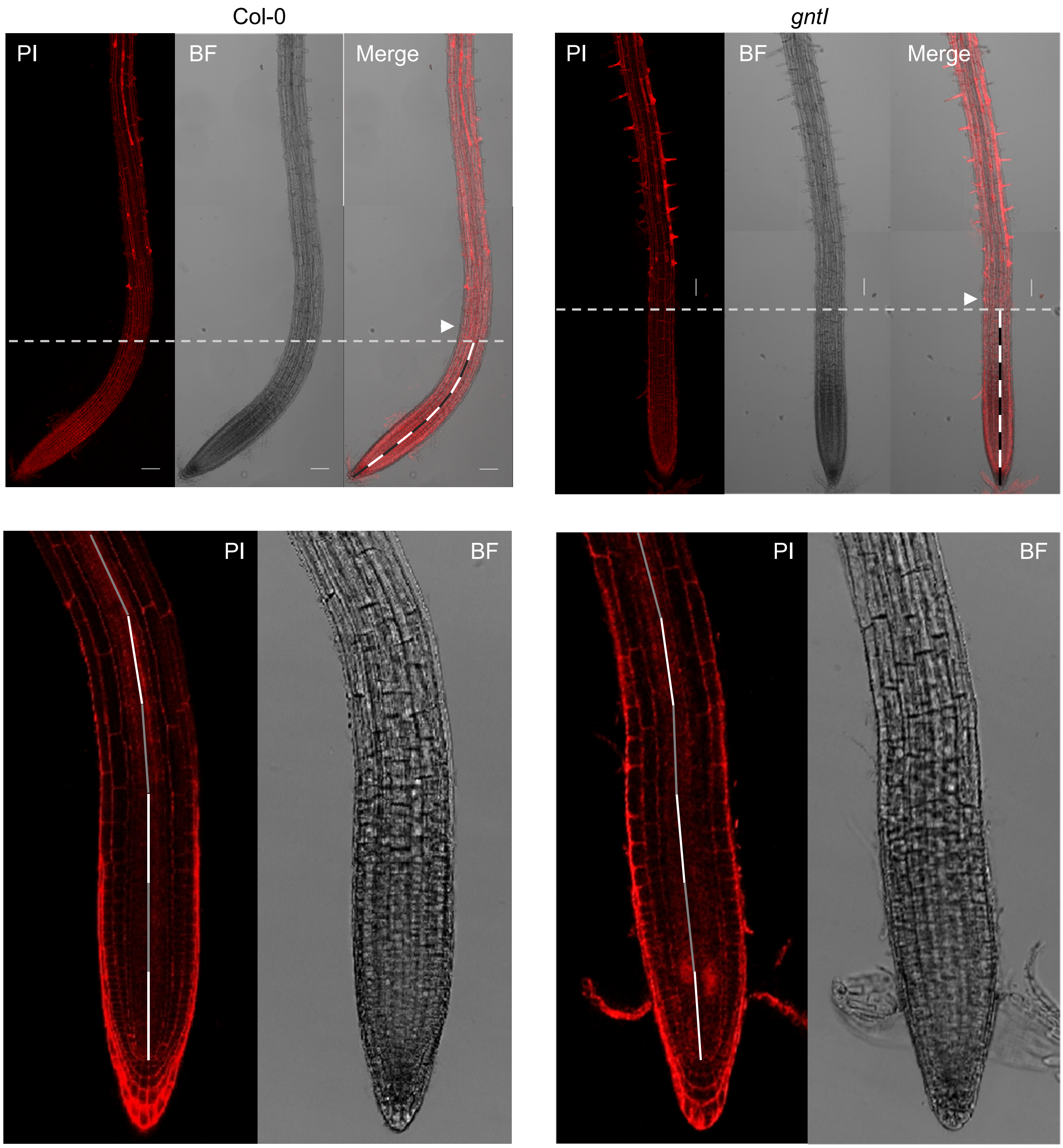

Figure S2. Picture of representative Col-0 and *gntl* roots stained with propidium iodide. Top, ten-day-old seedlings. White arrows mark the first visible root hair bulb. Dotted lines: first mm of the root tip. Bottom, meristematic zone of three-day-old seedlings. PI, propidium iodide; BF, bright field. Scale bars: 100 µm.

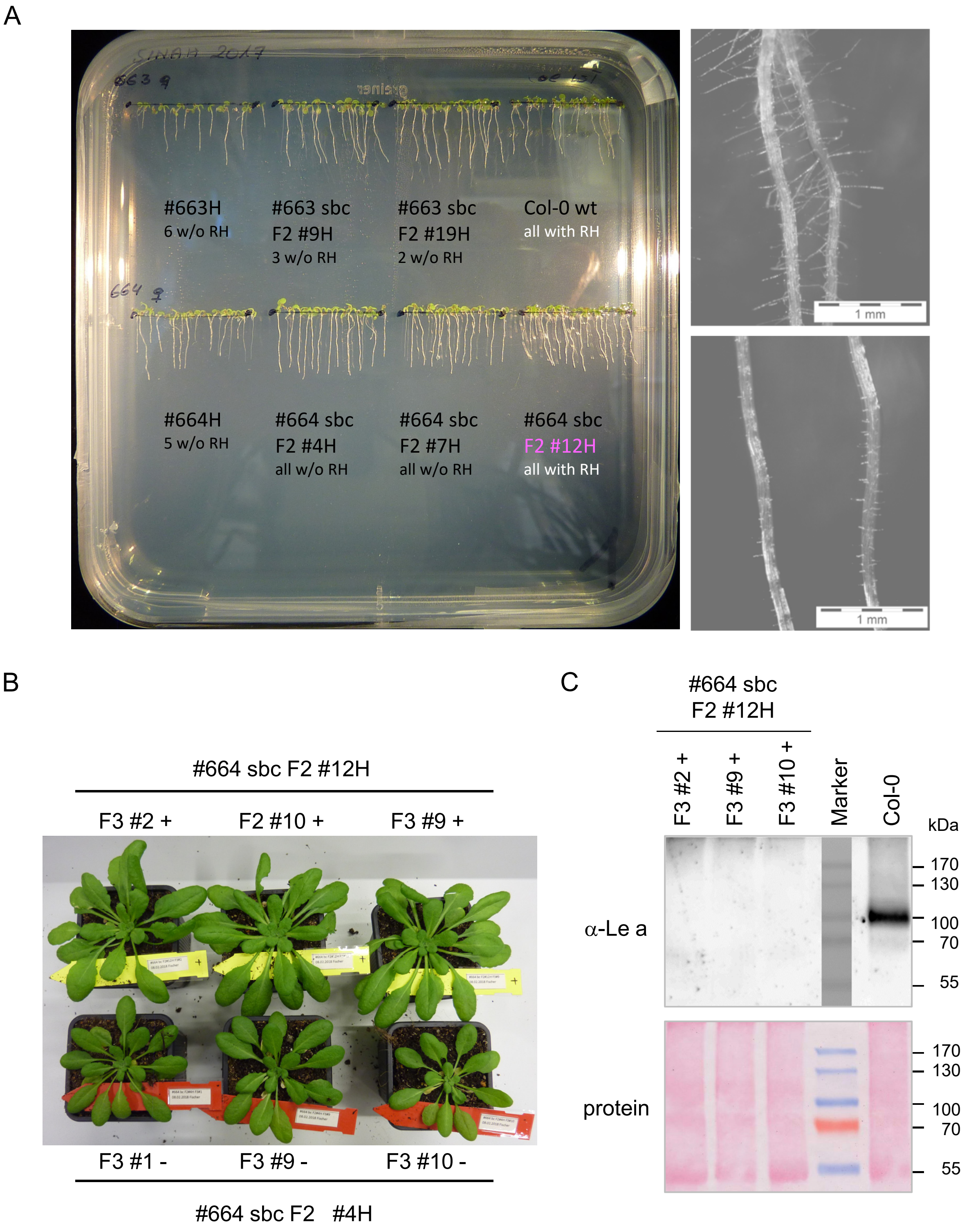

Figure S3. The *galt* line (SAIL_170_A08; Strasser et al. 2007) contained an unlinked RH mutation. A) Two homozygous *galt* plants (#663H and #664H) were backcrossed with wild type (Col-0 as pollen donor). F1 plants were checked by PCR for heterozygosity and allowed to self (sbc = selfed backcross). F2 plants were PCR tested for the *galt1-1* T-DNA insertion. Seeds of homozygous plants were surface sterilized, directly placed on square plates, sealed with Parafilm®, stratified, and grown vertically (left). Roots on plates were inspected for wild type-like RH (top right) or merely elongated RH (bottom right). B) F3 progeny of two F2 lines with (+) or without (-) RH. Note that the latter remain smaller upon growth in soil (bottom, red stickers). C) Immunoblot analysis of leaf extracts (pellet fractions). Using monoclonal rat antibodies (JIM84), lack of Lewis-a signals (α-Le a) was confirmed in *galt1* F3 plants of F2 #12H with RH (+). The Ponceau S-stained blot below is shown as loading reference (protein). Molecular masses are indicated in kDa (Marker, PageRuler Prestained Protein Ladder (Fermentas). Line F2 #12H was used for all further experiments.

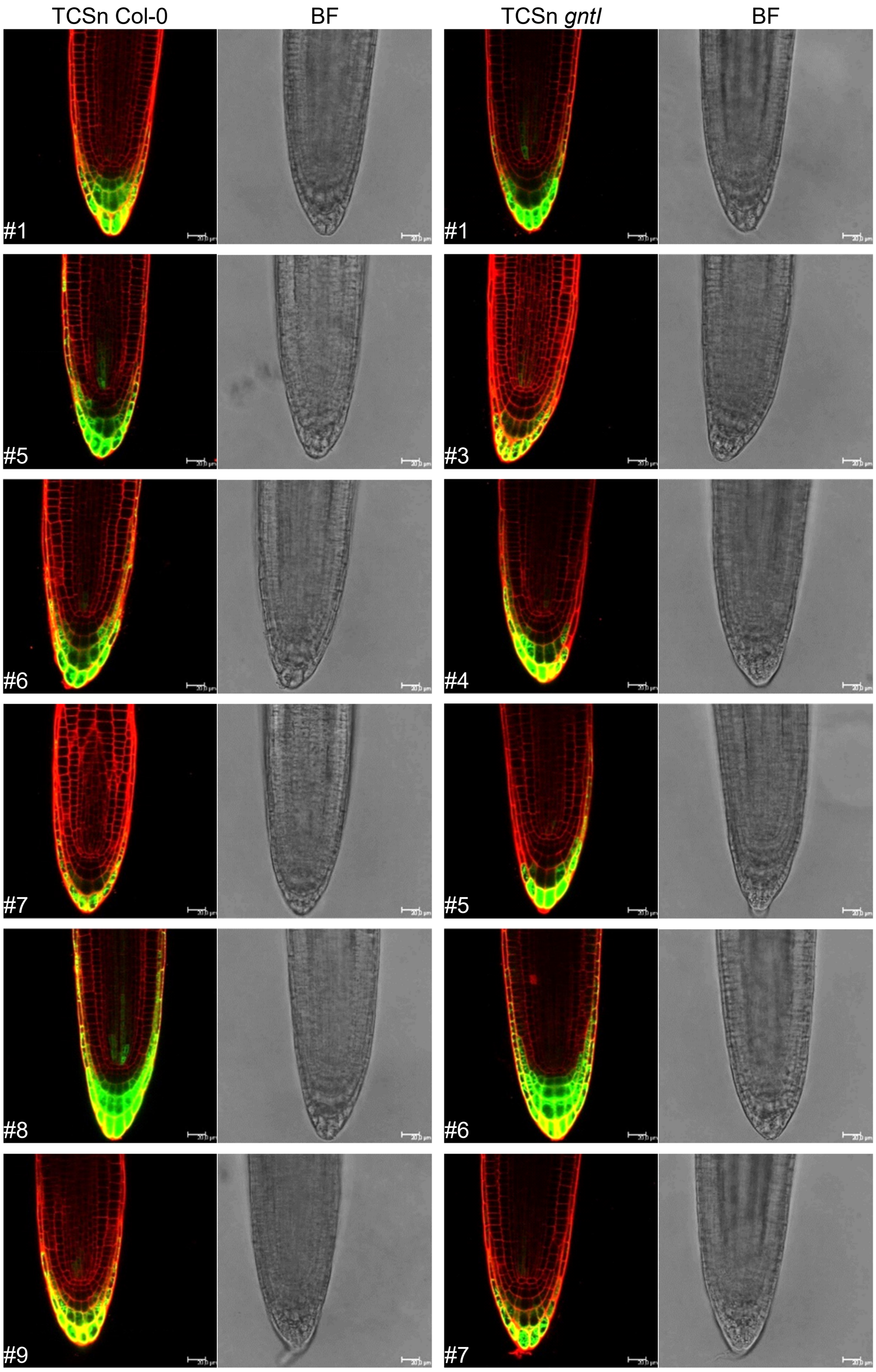

Figure S4. Root tip pictures of multiple independent *TCSn::GFP* (TCSn) lines (indicated by #) in Col-0 wild type and the *gntl* background taken in the same experimental setup shown in Figure 3. Scale bar: 20 µm. BF, bright field.

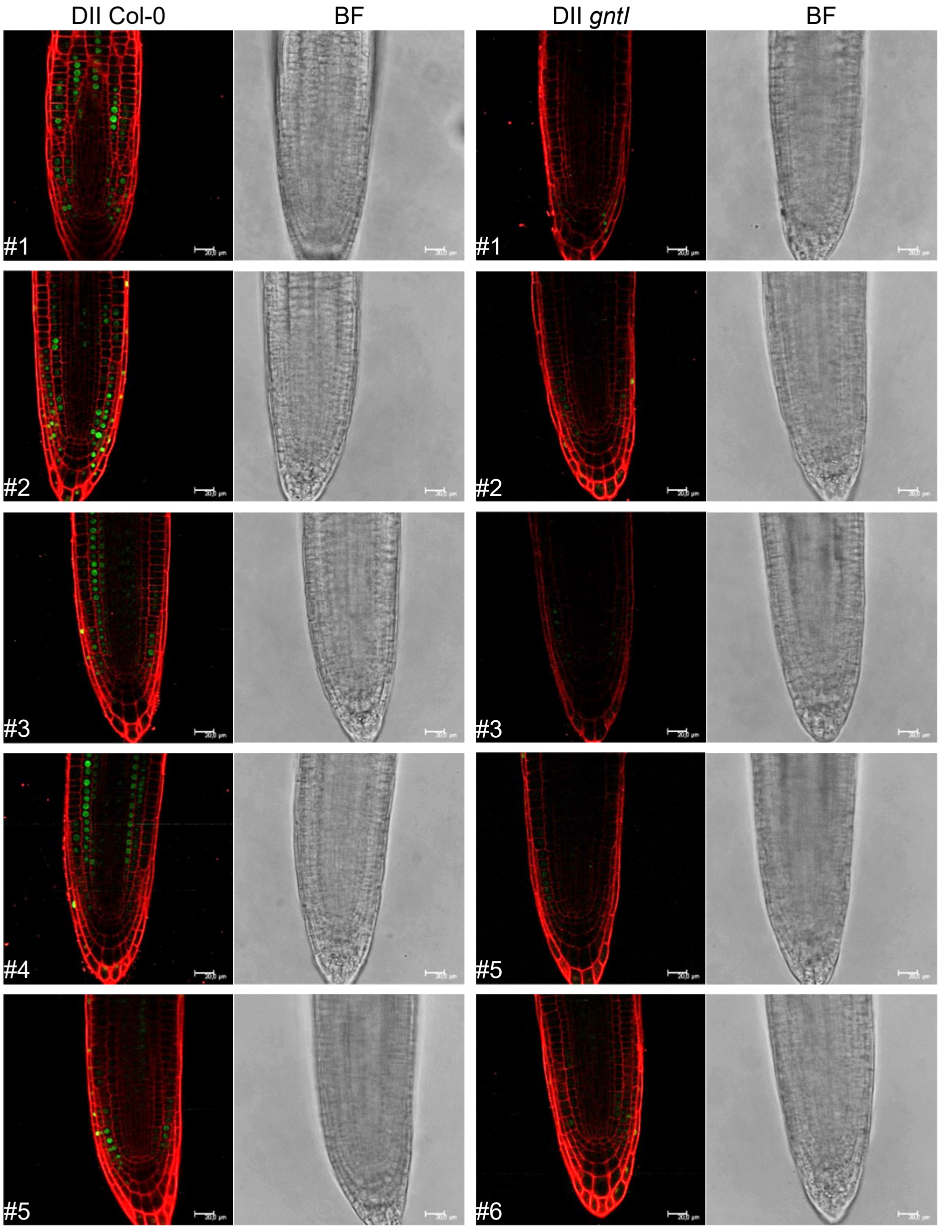

Figure S5. Root tip pictures of multiple independent *35S::DII-VENUS* (DII) lines (indicated by #) in Col-0 wild type and the *gntl* background taken in the same experimental setup shown in Figure 3. Scale bar: 20 µm. BF, bright field.

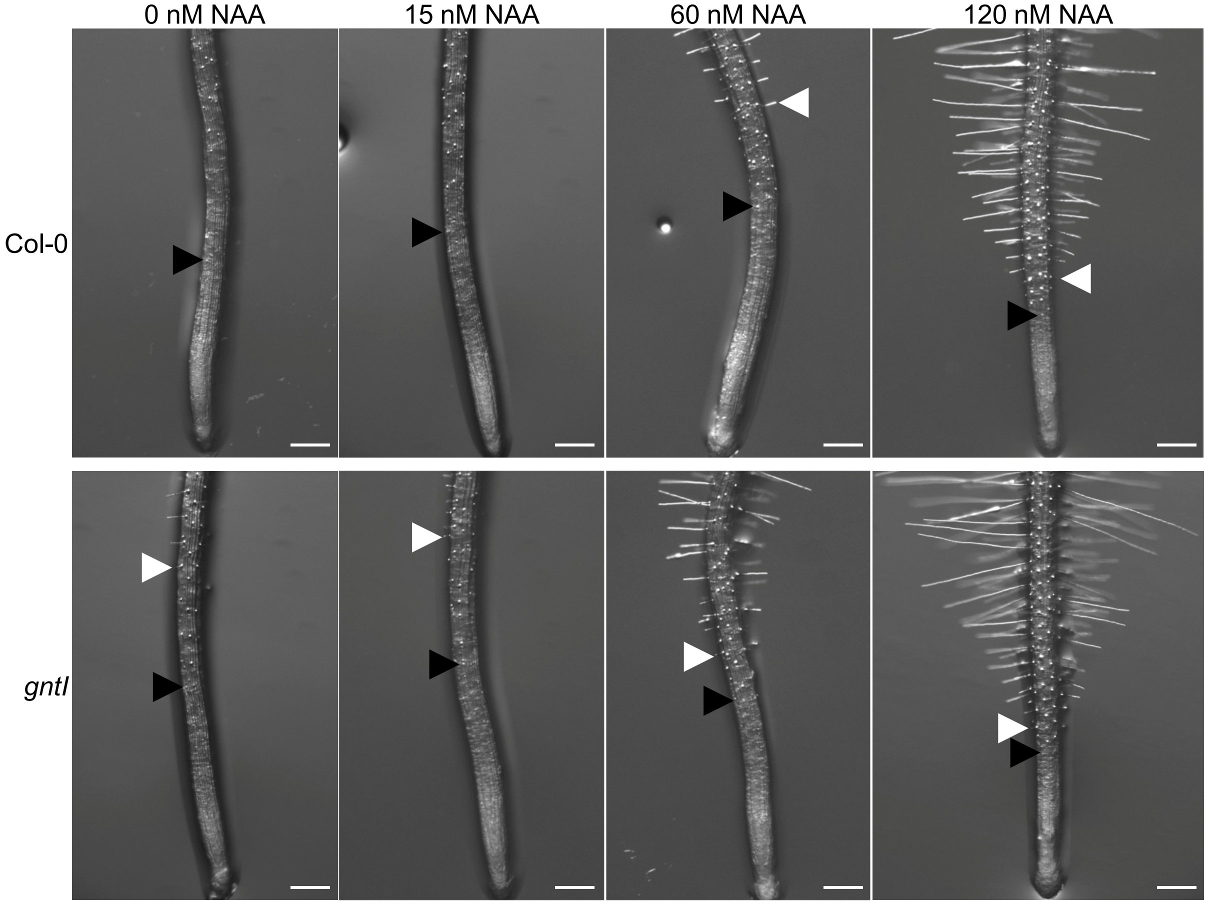

Figure S6. Pictures of representative Col-0 and *gntI* root tips taken for measurements depicted in Figure 4. Black arrows indicate the first general root hair while white arrows point to the first partially elongated root hair. Scale bar: 250 µm.

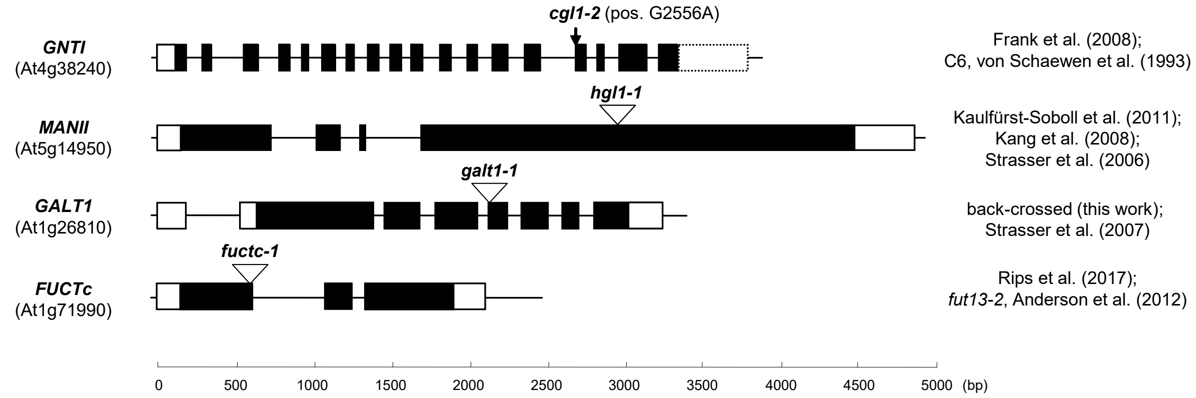

Figure S7. Schematic overview of mutant alleles used in this study. T-DNA insertions are indicated by a white triangle, while point mutations are displayed with a black arrow. Black boxes represent exon regions, black lines between exons display intron regions and white boxes 5' and 3' UTR, respectively.

Table S1. Genes found enriched in GO term 'cellular response to hypoxia' (GO:0071456) and sub GO terms.

Gene ID	Gene Name	Log2-fold expression (*gntl* vs. wild type)
AT5G57220	*P450 81F2, CYP81F2*	0.7297
AT1G26410	*Berberine bridge enzyme-like 6, FOX4*	0.7952
AT1G26380	*Berberine bridge enzyme-like 3, FOX1*	0.8968
AT1G14550	*Peroxidase 5, PER5*	0.7164
AT5G26920	*Calmodulin-binding protein 60 G, CBP60G*	0.6742
AT2G23270	*At2g23270*	0.8968
AT5G13320	*4-substituted benzoates-glutamate ligase, GH3.12*	0.8279
AT5G25250	*Flotillin-like protein 1, FLOT1*	0.6091
AT1G05880	*Probable E3 ubiquitin-protein ligase, ARI12*	0.6715
AT4G31970	*Xanthotoxin 5-hydroxylase, CYP82C2*	0.6538
AT5G41080	*Glycerophosphodiester phosphodiesterase, GDPD2*	0.4781
AT1G19250	*Probable flavin-containing monooxygenase 1, FMO1*	0.7658
AT4G34131	*UDP-glycosyltransferase 73B3, UGT73B3*	0.6294
